# Disordered topological graphs enhancing nonlinear phenomena

**DOI:** 10.1126/sciadv.adf9330

**Published:** 2023-04-05

**Authors:** Zhetao Jia, Matteo Seclì, Alexander Avdoshkin, Walid Redjem, Elizabeth Dresselhaus, Joel Moore, Boubacar Kanté

**Affiliations:** ^1^ Department of Electrical Engineering and Computer Sciences, University of California, Berkeley, Berkeley, CA 94720, USA.; ^2^ Department of Physics, University of California, Berkeley, Berkeley, CA 94720, USA.; ^3^ Materials Sciences Division, Lawrence Berkeley National Laboratory, 1 Cyclotron Road, Berkeley, CA 94720, USA.

## Abstract

Complex networks play a fundamental role in understanding phenomena from the collective behavior of spins, neural networks, and power grids to the spread of diseases. Topological phenomena in such networks have recently been exploited to preserve the response of systems in the presence of disorder. We propose and demonstrate topological structurally disordered systems with a modal structure that enhances nonlinear phenomena in the topological channels by inhibiting the ultrafast leakage of energy from edge modes to bulk modes. We present the construction of the graph and show that its dynamics enhances the topologically protected photon pair generation rate by an order of magnitude. Disordered nonlinear topological graphs will enable advanced quantum interconnects, efficient nonlinear sources, and light-based information processing for artificial intelligence.

## Teaser

Nonlinear processes in topological systems are enhanced by synthetic structural disorder.

## INTRODUCTION

Disorder in two-dimensional electronic systems leads to a wide range of topological phenomena, including integer and fractional quantum Hall effects, in which impurities resulting from the sample fabrication process break the degeneracies of Landau levels and localize the wave functions at almost all energies (*1*–*4*). Such localization is the direct cause of quantized plateaus in the Hall conductance, as the localized states do not contribute to the particle transport and the quantization plateaus would cease to exist in an ideal clean sample (*1*, *5*–*6*).

The hallmark of topological phenomena in two-dimensional finite-size systems is the appearance of a transport channel that is robust to disorder (*7*). The robustness to disorder has been investigated and exploited in many wave-based phenomena including photonics, microwaves, acoustic, and plasmonics to conceive devices potentially more robust to manufacturing imperfections that usually degrade the performance of classical systems and cause decoherence in quantum systems (*3*). Topological transport, occurring along boundaries, can be unidirectional and immune to backscattering, provided that the disorder is not strong enough to close the mobility gap. The unique robustness of topological transport has led to advanced device concepts, including topological delay lines, topological lasers, topological frequency combs, and topological quantum light sources (*8*–*16*). However, most attention has focused on the treatment of potential disorder, i.e., on spatially local random potentials. A different type of disorder, known as structural disorder, has long existed in nature, for example, in amorphous silicon. In this noncrystalline form of silicon, the local connectivity is preserved, since each silicon atom is bonded to four adjacent neighbors, but long-range structural order is lost. Nevertheless, a mobility gap in amorphous matter has been observed (*17*–*18*). More recently, topological phenomena have been observed in nonperiodic systems (*19*–*24*). These findings lead to a natural question to ask: beyond preserving topological properties, can disordered topological systems outperform their periodic counterpart? We propose a structurally disordered system that exhibits a nontrivial topological phase, characterized by a nonuniform synthetic magnetic flux. We show that, in the presence of nonlinearities, the structurally disordered system prevents the ultrafast leakage of energy from topological modes to bulk modes, enhancing nonlinear phenomena. As an example, we demonstrate that the longer confinement of light can lead to an order of magnitude increase in the generation rate of correlated photon pairs compared to periodic topological platforms.

## RESULTS

### Disordered linear topological graph

**Fig. 1.**
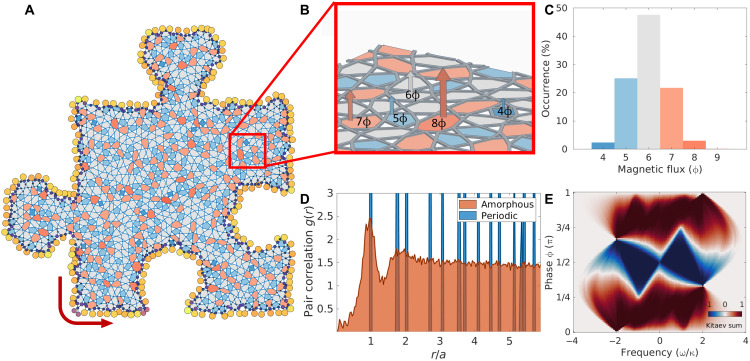
Principle and design of amorphous topological graphs. (**A**) Sketch of an amorphous topological graph. The local coordination number *z* is preserved (*z* = 4), while the graph connectivity is different from the periodic counterpart. Different colors indicate polygonal plaquettes with different number of sides. (**B**) Zoomed-in view of (A), showing the presence of a nonuniform magnetic field flux. The labels quantify the magnetic flux across each polygonal plaquette, which is equal to the overall hopping phase acquired by a photon through a round-trip around the plaquette. (**C**) Distribution of polygonal plaquettes with *N* ≥ 4 sides for the graph in (A). The periodic lattices only have hexagons with *N* = 6. (**D**) Pair correlation function *g*(*r*) of the amorphous structure in (A), compared to the periodic lattice. The amorphous structure lacks long-range order, as evidenced by the flattened pair correlation function at longer distances *r*/*a*. (**E**) Topological phase diagram for the amorphous structure. The color represents the Kitaev sum calculated over all the modes below different values of the cutoff frequency ω/κ, for different hopping phases ϕ between adjacent vertices. The result is averaged over 20 realizations of disorder.

**Fig. 2.**
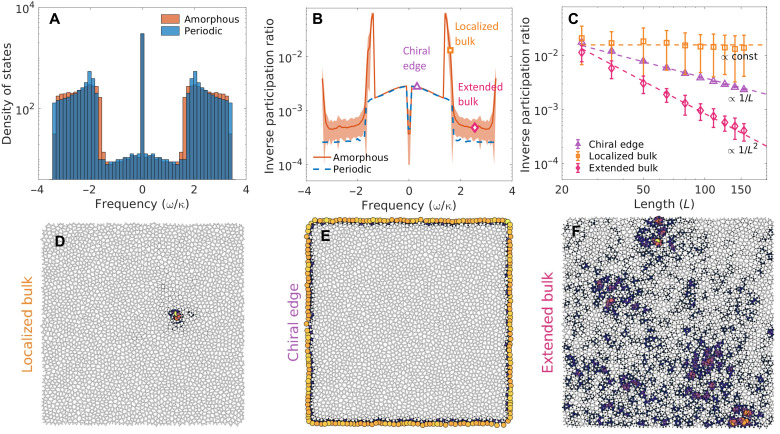
Scaling of modes in periodic and amorphous topological graphs. (**A**) Density of states for the amorphous and periodic topological graphs, for a hopping phase ϕ = π*/*2. (**B**) Inverse participation ratio for the eigenmodes of the periodic and amorphous graphs. Three types of modes [chiral edge (CE), localized bulk (LB), and extended bulk (EB)] are observed with distinct IPRs. The shaded area represents the standard deviation for 20 realizations of structural disorder. (**C**) Scaling of the IPR with the graph size *L*, for different modes. The results obtained by diagonalizing the linear Hamiltonian in Eq. 1 agree well with the theory, and the IPR of modes scales as a constant, 1/*L*, and 1/*L*^2^ for LB, CE, and EB modes, respectively. The error bars are obtained by averaging over 20 realizations. (**D** to **F**) Intensity profiles of three representative modes, classified as LB (D), CE (E), and EB (F), showing different localization features. The local light intensity is proportional to the size of the circles at each site, and it is visualized by thermal-like fill color.

The proposed nonlinear amorphous graph, presented in Fig. 1, is constructed by kagomizing a Voronoi diagram obtained from a disk-sampled set of points (Supplementary Materials) (*23*). The result is a collage of polygonal plaquettes, each having three to nine sides, as sketched in Fig. 1A. Adjacent vertices are then coupled by a directional hopping with uniform magnitude κ and phase factor *e*^*i*ϕ^, shown as the graph edges in the sketch in Fig. 1A. The linear tight-binding Hamiltonian can be written as

$${\hat{H}}_{0}=\sum_{i}\omega_{0}{\hat{a}}_{i}^{†}{\hat{a}}_{i}-\kappa \sum_{\left\langle i,j\right\rangle }(e^{-i\phi }{\hat{a}}_{i}^{†}{\hat{a}}_{j}+e^{i\phi }{\hat{a}}_{j}^{†}{\hat{a}}_{i})$$ (1)

where ${\hat{a}}_{i}^{†}$ (${\hat{a}}_{i}$) creates (annihilates) a particle on the *i*th site, ω_0_ is the natural on-site frequency, and <*i,j*> restricts the summation to pairs of nearest neighbors. The additional hopping phase ϕ, which can be tuned in a photonic implementation (Supplementary Materials), can be interpreted as the Peierls phase resulting from the presence of a synthetic magnetic field. In the model considered here, the synthetic magnetic flux across a polygonal plaquette of the graph depends on the number of edges of the plaquette. Specifically, within each triangular plaquette (white in Fig. 1A), a constant synthetic magnetic field flux of −3ϕ is accumulated. In contrast, as sketched in Fig. 1B, the synthetic magnetic field threading each polygonal plaquette with at least four sides is different, and it can vary from 4ϕ to 9ϕ, proportionally to the number of sides of the polygonal plaquette. Therefore, unlike the anomalous quantum Hall systems that feature a uniform magnetic flux inside hexagonal plaquettes (*25*–*26*), the proposed amorphous system has a nonuniform magnetic flux across different plaquettes based on the real-space connectivity. The statistical distribution of the magnetic flux per plaquette is shown in Fig. 1C, and it can be controlled by changing the filling ratio in the original random disk sampling process (Supplementary Materials). By preserving the local connectivity, the generated structure inherently has short-range order but lacks long-range order. This can be inferred from Fig. 1D, where a flattened pair correlation function between vertices is observed, unlike periodic structures that exhibit characteristic sharp peaks. Despite the structural disorder, the system shows the hallmarks of nontrivial topology. By controlling the hopping phase, the system undergoes a topological phase transition that opens a nontrivial mobility gap. The topological nature of such a gap can be verified by calculating a topological marker known as “Kitaev sum,” shown in Fig. 1E (*23*, *27*), whose value at some frequency ω expresses the accumulated Chern number from all the bands below the chosen frequency. The complex hopping term *e*^−*i*ϕ^ allows for a selective tuning of the Kitaev sum across a phase boundary between −1 and +1, when ϕ is set to a value between 0 and π. A nonzero topological marker in the gap implies that a finite-size system will exhibit chiral topological edge states, unidirectionally guided along the physical edge. These states, marked by a low density of states in the topological gap (Fig. 2A), emerge in both periodic and amorphous systems and are robust to on-site potential disorder as long as the disorder strength is not comparable to the bandgap (Supplementary Materials). However, while a strong enough on-site potential disorder will eventually overcome the topological protection of the edge states, an increasing degree of structural disorder will not affect the topological properties of the system, i.e., the edge states are topologically protected irrespective of the degree of structural disorder (*24*). The regions with a higher density of states in Fig. 2A correspond to bulk bands, including a flat band at zero frequency. We classified the eigenstates ψ by calculating their inverse participation ratio (IPR), defined as $\mathrm{IPR}(\psi )=\sum_{i}{|\psi_{i}|}^{4}/{|\sum_{i}{|\psi_{i}|}^{2}|}^{2}$, whose scaling law with respect to the lattice size is a measure of the localization of the eigenstates within finite-size systems. The IPR presented in Fig. 2B shows that our amorphous structure features three types of eigenmodes, which are chiral edge (CE) modes, localized bulk (LB) modes, and extended bulk (EB) modes. The localization and the scaling properties of the modes with the size of amorphous graphs are summarized in Fig. 2C. For a two-dimensional graph with disk sampling domain area *L*^2^, the IPR scales as a constant for LB modes, as 1/*L* for CE modes, and as 1/*L*^2^ for EB modes. Intensity profiles of three representative modes are shown in Fig. 2 (D to F). The IPRs of the CE modes scale like their periodic counterparts, indicating the existence of topological edge transport channels, while the LB modes, which are a unique feature of the amorphous system, originate from the presence of structural disorder. The LB modes stand out as characteristic peaks in the IPR of Fig. 2B, occurring in the vicinity of the band edges, and they are responsible for the mismatch between the density of states of periodic and amorphous systems (Fig. 2A). The EB modes, which are spatially delocalized, feature low IPRs. The introduction of structural disorder has therefore dramatically changed the localization nature of the bulk modes, introducing the LB modes near the band edges. In addition, the remaining bulk modes are more localized, while the topological nature of the system is preserved.

### Disordered nonlinear topological graph

We now consider the nonlinear dynamics of the amorphous topological graph by including multiparticle interactions. As a prototypical example relevant for a wide class of systems, we will focus here on local two-particle interactions, such as the ones that occur between photons in a Kerr medium. The full Hamiltonian $\hat{H}$ describing the nonlinear graph is written by adding a term $\hat{V}$ to the linear Hamiltonian ${\hat{H}}_{0}$

$$\hat{H}={\hat{H}}_{0}+\hat{V}$$ (2)

where

$$\hat{\;V}=U_{0}\sum_{i}{\hat{n}}_{i}^{2},\;{\hat{n}}_{i}={\hat{a}}_{i}^{†}{\hat{a}}_{i}$$ (3)

**Fig. 3.**
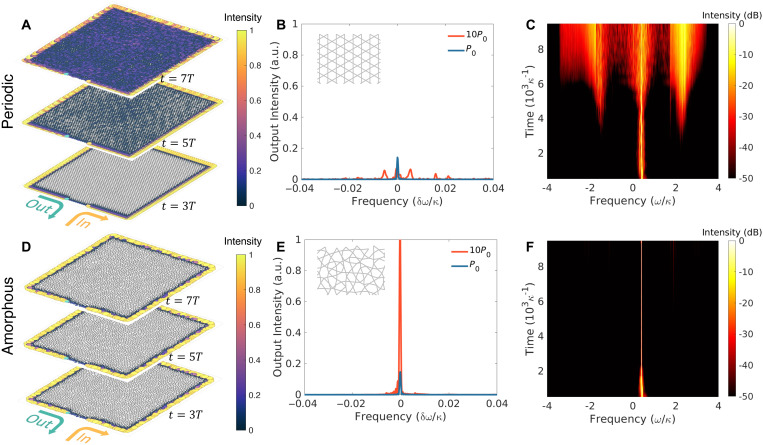
Enhanced nonlinear topological transport in amorphous graphs. (**A** and **D**) Snapshots of the real-space intensity distribution in periodic (A) and amorphous (D) structures, following an initial pulse excitation injected from the input channel, taken at times 3*T*, 5*T*, and 7*T*, where *T* = 1000 κ^−1^ is approximately the time it takes for the signal to travel from the input to the output port. Energy leaks from the edge to the bulk modes in the periodic graph, while it remains confined along the edge in the amorphous graph for longer times. (**B** and **E**) Power spectrum at the output channel for two different input powers *P*_0_ and 10*P*_0_, for the periodic (B) and amorphous (E) graphs. The additional peaks in the periodic graph spectrum at 10*P*_0_ pumping correspond to the coupling between adjacent CE modes. The insets show a zoomed-in view of periodic (B) and amorphous (E) graphs. a.u., arbitrary units. (**C** and **F**) Time evolution of the power spectrum after injecting an initial signal at an edge mode frequency, obtained via a short-time Fourier transform. In the amorphous case (F), the energy couples to other edge modes or bulk modes at a slower rate compared to the periodic case (C).

In Eq. 3, *U*_0_ is a material-dependent strength of the nonlinearity and ${\hat{n}}_{i}$ is the particle number operator at site i. The time dynamics of the nonlinear system is obtained by explicitly integrating the time-evolution equations. The periodic and the amorphous topological systems are both driven with the same amplitude, which is spectrally peaked within the topological bandgap. Snapshots of the intensity distribution at different times *t* are shown for the periodic (Fig. 3A) and amorphous (Fig. 3D) graphs. The energy of the propagating excitation is confined near the boundary at early time stages in both cases (*t* < 3*T*), where *T* = 1000 κ^−1^ is approximately the time the signal takes to travel from the input to the output port. In the periodic case (Fig. 3A), the excited CE modes couple to other CE modes and then leak toward the bulk as the EB modes are fed (*t* = 5*T*). This contrasts with the amorphous case, where energy is confined within the CE modes at *t* = 5*T*, as shown in Fig. 3D. The difference in energy transport in the presence of nonlinearity is quantified by probing the transmission at the output port for different injected power levels, as shown in Fig. 3 (B and E). For a relatively weak pumping power *P*_0_, the transmission spectrum is similar in the periodic and amorphous systems. As the pumping power increases, the edge transport channel breaks down in the periodic system due to the nonlinearity-induced coupling, while the transmission in the amorphous system maintains CE propagation, leading to an almost 10-fold increase in the peak power difference at the output. The stronger coupling between adjacent CE modes in the periodic case is confirmed by the presence of additional side peaks in the transmission spectrum. The evolution of the two systems in the time-frequency domain, obtained via a short-time Fourier transform, is presented in Fig. 3 (C and F). In the periodic system, at early times, the initialized CE mode couples to the spectrally closest CE modes, resulting in frequency broadening. As time evolves, the coupling between the excited CE modes and EB modes, induced by the presence of the nonlinearity, ignites bulk modes, which in turn excite other EB and CE modes. The result, at long times, is a markedly broadened spectrum. The amorphous system in Fig. 3F, however, shows a notably different behavior, displaying both reduced oscillations between adjacent CE modes as well as a suppressed appearance of additional modes in the spectrum, achieving an almost unperturbed propagation for much longer times.

## DISCUSSION

The increased isolation that the injected CE mode experiences in the amorphous system can be understood as an interplay of different mechanisms. First, the broken periodicity resulting from the introduction of structural disorder precludes us from identifying a well-defined momentum for the EB modes, hampering the fulfillment of phase-matching conditions for the nonlinearity-induced coupling, and suppressing the initial oscillations between CE modes. Second, according to Fermi’s golden rule, the initially excited bulk modes will be located around the peak of density of states in Fig. 2A, close to the band edges. These modes have an EB nature in the periodic system, but with the introduction of structural disorder, some of them become LB modes in the amorphous system (*28*). The localization of LB modes then delays the nonlinearity-induced propagation from CE to EB modes, with the latter being excited at much later times. The eventual propagation of the signal to the EB modes is delayed by the introduction of structural disorder, but not completely suppressed, as an inevitable consequence of the presence of the nonlinearity.

**Fig. 4.**
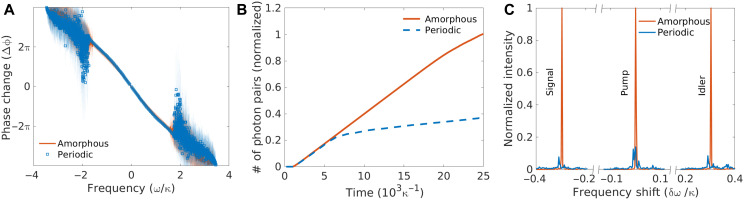
Disorder-enhanced topological light generation. (**A**) Effective dispersion relation of amorphous and periodic edge states, obtained by plotting the average phase change between consecutive triangular plaquettes along the edge against the frequency of each eigenmode. For the amorphous system, dispersion broadening is observed due to the aperiodic variation of the phase at different positions along the edge state. Note that, for bulk modes, the quasi-linear phase change breaks down due to the lack of well-defined transport channels. (**B**) Time evolution of the total number of generated photon pairs. The photon pair generation rate (slope of the curve) decreases in the periodic system as the signal/idler modes couple to the EB modes, while the generation rate remains high for longer time in the amorphous system. (**C**) Example of the normalized spectra of photon pairs generated via a pump at frequency ω_p_ = 0.4 κ. The spectra are centered at the pump frequency.

The spatial and spectral energy confinement in the presence of nonlinearity can be used, for example, to enhance the efficiency of quantum topological photon pair generation via spontaneous four-wave mixing in optical systems (*29*–*30*). In this nonlinear four-photon process, a pump signal is injected into the system via an input port, and the correlated photon pairs generated within the topological bandgap are guided toward the output port along the boundaries. The positions of input and output ports are chosen as shown in Fig. 3 (A and D) to increase the distance traveled by the pump signal, thereby maximizing the photon pair generation efficiency. The system is described by the following Hamiltonian (*29*, *31*–*32*)

$$H_{SI}=H_{0}^{\left(S\right)}+H_{0}^{\left(I\right)}+\sum_{i}\chi_{i}{\hat{a}}_{i}^{{\left(S\right)}^{†}}{\hat{a}}_{i}^{{\left(I\right)}^{†}}$$ (4)

where the last term creates correlated pairs of photons, called signal (*S*) and idler (*I*), at a site-dependent rate $\chi_{i}=\chi_{0}\;{\hat{a}}_{i}^{\left(P\right)}{\hat{a}}_{i}^{\left(P\right)}$, which depends both on the pump (*P*) strength and on the optical nonlinearity χ_0_. The terms $H_{0}^{\left(S/I\right)}$ are the bare Hamiltonians of the signal and idler photons, respectively. The effectiveness of this process typically relies on working with a quasi-linear dispersion to satisfy both energy and momentum conservation. As shown in Fig. 4A, the topological dispersion in our system fulfills this requirement. The photon pair generation efficiency is presented in Fig. 4B. Both the periodic and the amorphous system generate a similar number of photon pairs at early times. At later times, however, the efficiency of the periodic system drops due to the increased coupling between CE and EB modes, while the amorphous system remains efficient. The enhanced spectra of signal, pump, and idler are presented in Fig. 4C. In the periodic case, the self-modulation of the pump leads to a reduced lifetime of the edge modes at the excitation frequencies; hence, the photon pair generation rate is also reduced, while in the amorphous case, the pumped edge states can generate more photon pairs.

We proposed and demonstrated an amorphous topological platform (graph) to enhance nonlinear phenomena. The disordered topological graphs are based on the control of a synthetic magnetic field threading through different polygonal plaquettes and leading to a nonuniform flux that gives rise to the nontrivial topology of the graph. The nonlinear responses of such amorphous topological graphs outperform their periodic counterparts, owing to the emergence of LB modes and to a reduced phase matching, enabling the ultrafast guiding of energy, protected against leakage. The accumulation of energy in the topologically protected channel enhances the generation of photon pairs. The proposed scheme for enhancing nonlinear responses via the introduction of structural disorder in topological systems will have a broad range of applications, including devices for robust light-based information processing and computing.

## MATERIALS AND METHODS

### Generation of the amorphous topological graph

The amorphous graphs are generated via a three-step procedure to maintain a fixed coordination number while implementing the structural disorder. First, disk sampling with a random seed is used to introduce disorder in the system. Then, the centers of the disks are used to generate a Voronoi graph with a fixed coordination number of 3. The amorphous Kagome graph is finally constructed by connecting the centers of adjacent edges around each vertex of the Voronoi diagram. The generated Kagome graph has a coordination number of 4, which is the same as that of a periodic Kagome lattice. In the disk sampling process, the filling ratio can be used to control the degree of disorder. In the amorphous structures simulated in this work, a filling ratio of η = 0.45 is used with a domain size of *L*/*r* = 250 in the disk sampling step.

### Calculation of the topological index

The topology of the amorphous graph is explored via the Kitaev sum. The graph is first triparted into three adjacent spatial regions A, B, and C that share a common vertex at the center of the graph and that cover 1/4 of its area. Then, the Kitaev sum at frequency ω*_c_* is calculated as

$$\nu (P)=12\pi \sum_{i\in A}\sum_{j\in B}\sum_{k\in C}(P_{ij}P_{jk}P_{ki}-P_{ik}P_{kj}P_{ji})$$

where *P* is the projection operator onto the eigenmodes below ω*_c_* and *i*, *j,* and *k* are site indices.

### Time-domain simulations

The time-domain simulations were performed on MATLAB via explicit integration of the semiclassical evolution of the particle fields, using a fourth-order Runge-Kutta scheme to ensure numerical stability. The four-wave mixing process was simulated in the undepleted pump approximation, under which the fields evolve via a time-dependent Hamiltonian that can be represented as the following matrix (*31*)

$$H_{SI}(t)=\left[\begin{array}{c} H_{0}^{\left(S\right)}\;\;\;\;C(t)\\ C^{†}(t)\;\;\;\;H_{0}^{(I)†} \end{array}\right]$$

where *C_ij_*(*t*) = χ*_i_*(*t*)δ*_ij_* and the state vector is expressed as $\psi ={\left[a_{1}^{\left(S\right)},\ldots ,a_{N}^{\left(S\right)},a_{1}^{(I)†},\ldots ,a_{N}^{(I)†}\right]}^{T}$. The outputs are Fourier-transformed to obtain the spectral information.

## Supplementary Materials

### This PDF file includes:

Supplementary Text Figs. S1 to S6 References
