# Supplementary material for: Disordered topological graphs enhancing nonlinear phenomena
Source: Sci Adv. 2023 Apr 5;9(14):eadf9330. doi: 10.1126/sciadv.adf9330 (PMC10075993; doi:10.1126/sciadv.adf9330)
Supplement: Supplementary file 1 — Supplementary Text Figs. S1 to S6 References [file sciadv.adf9330_sm.pdf]

Science Advances
AAAS

Supplementary Materials for

Disordered topological graphs enhancing nonlinear phenomena

Zhetao Jia *et al.*

Corresponding author: Boubacar Kanté, bkante@berkeley.edu

Sci. Adv. **9**, eadf9330 (2023)
DOI: 10.1126/sciadv.adf9330

This PDF file includes:

Supplementary Text
Figs. S1 to S6
Reference

S.1. PERIODIC KAGOME LATTICE AND BAND STRUCTURE

To investigate the proposed amorphous topological graph, we first study its periodic counterpart.[33] In Fig. S1, a finite-size periodic kagome lattice is shown. Its unit cell is highlighted within the red hexagon, with arrows indicating a directional hopping phase $e^{-i\phi}$. The Hamiltonian of the system is of the same form as Eq. 1 in the main text, but the connectivity is different in periodic and amorphous systems. The difference is encoded in the positions of nonzero matrix elements in the Hamiltonian of the tight-binding model, and cannot be eliminated by a permutation of the basis elements, i.e. the two graphs are non-isomorphic. In the periodic system, the hopping phase ϕ determines the band structure, and opens a topological gap when the hopping phase is tuned from $\phi = 0$ to $\phi = \pi/2$. The nontrivial topological properties can be illustrated by the Chern number, calculated by integrating the Berry curvature in momentum space. The calculated topological indices of three bands are labeled in Fig. S1c, with -1, 0, 1 from lower to higher bands, respectively.

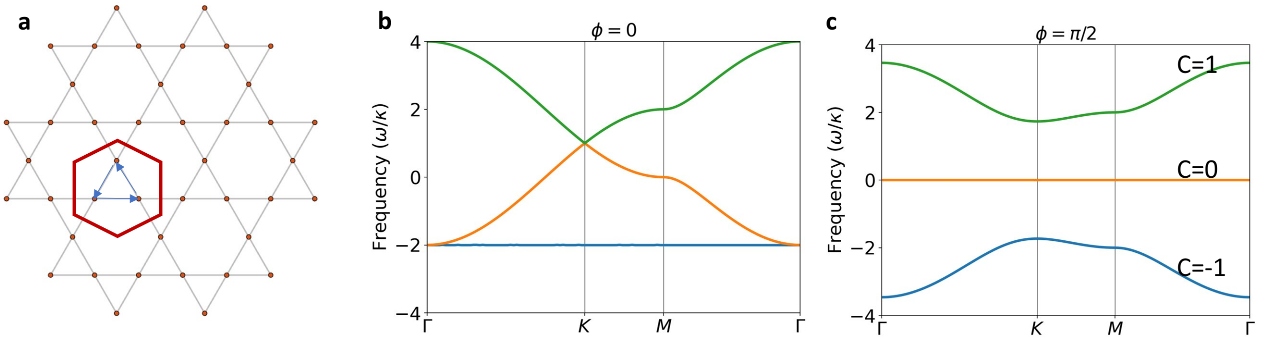

Fig. S1 | Topological kagome lattice. **a**, Structure of a finite-size kagome lattice with a directional hopping phase. The unit-cell, containing three sites, is highlighted by the red hexagon. **b**, Band diagram of a kagome lattice with a null directional hopping phase ($\phi = 0$), where the coupling between sites is real. **c**, Band diagram of a topological kagome lattice with $\phi = \pi/2$, which corresponds to a complex hopping $e^{-i\phi} = -i$. Topological bands with nonzero Chern numbers appear.

S.2. GENERATION OF THE TOPOLOGICAL AMORPHOUS GRAPH

The topological amorphous graph is generated by tessellation and local triangularization of randomly sampled points in two-dimensional space.[23] The initial random points can be generated, for example, with disk sampling (Fig. S2a) in a domain with area L^2 and periodic boundary conditions. The filling ratio η, defined as the ratio of area covered by disks over the area of the sampling domain, is the parameter used to control the strength of structural disorder. The Voronoi diagram is used to partition space, and create a graph with a fixed coordination number $N = 3$ (Fig. S2b). The amorphous graph is created by a kagomization process, in which the centers of the edges sharing the

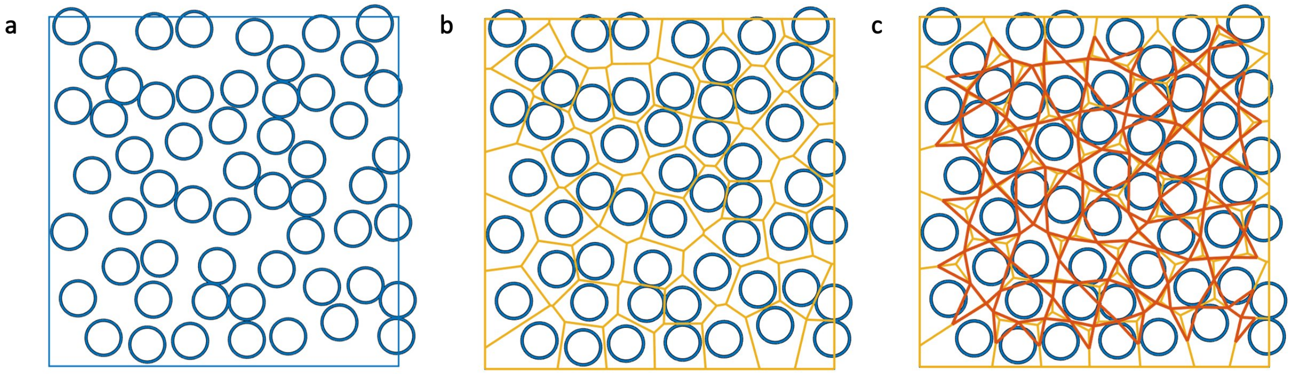

Fig. S2 | Amorphous graph generation procedure. **a**, Disks are randomly sampled within the domain with periodic boundary conditions. Each disk has a fixed radius and does not overlap with its neighbors. **b**, Voronoi diagram is generated based on the disk centers. **c**, The centers of edges sharing to the same vertices are connected to generate a kagome-like graph.

same vertices are connected. The resulting graph has a local coordination number ($N = 4$) in Fig. S2c. Because of the coordination number $N = 3$ for the Voronoi diagram, the generated amorphous graph consists of local triangles around the vertices of the Voronoi diagram, but the tessellated regions consist of polygons with different number of sides. The periodic lattice in Fig. S1a can also be generated by the same procedure, with the difference that, instead of starting from a random point set, we use a triangular lattice. The triangular lattice arrangement, indeed, is the most compact configuration achievable via disk sampling, characterized by a maximum filling ratio $\eta_{max} = 0.9069$. The amorphous graphs are generated with a fixed filling ratio $\eta = 0.45$ for the disk sampling process. To make sure the sampling can be achieved within reasonable simulation time, the amorphous graph used in Fig. 3 and 4 has a domain size $L/r = 250$, where r is the radius of the disk. To compare with the periodic system, the total number of sites is controlled. Due to the randomness of the disk sampling process, the exact number of sites may differ, but the difference is less than 1% among realizations.

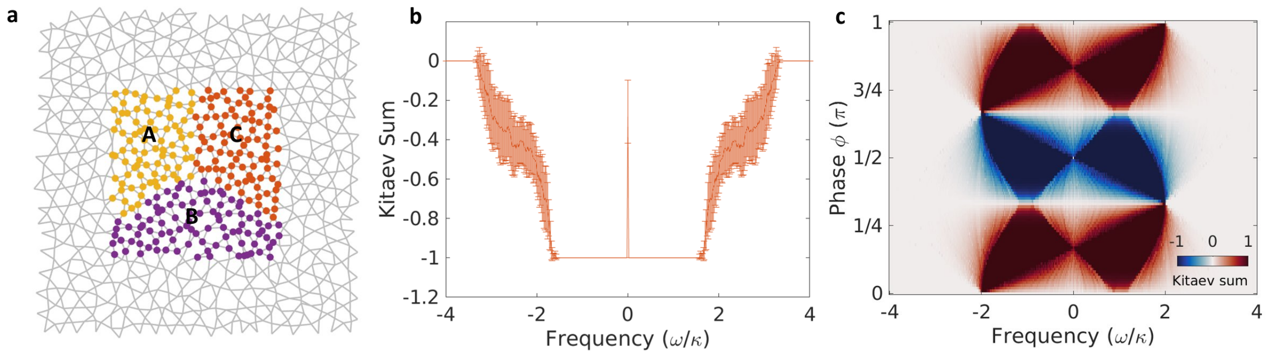

Fig. S3 | Kitaev sum calculation. **a**, Calculation of the Kitaev sum in the amorphous structure. To calculate the local topological index, the integration region is subdivided into three sections, marked here with different colors. **b**, Kitaev sum averaged over 20 random realizations of a graph with a hopping phase $\phi = \pi/2$. **c**, Kitaev sum calculated on a finite-size periodic lattice.

S.3. CHERN NUMBER CALCULATION IN THE AMORPHOUS TOPOLOGICAL GRAPH

The topology of the graph is probed by the Kitaev sum, a topological index defined as[23,27]

$$\nu(P) = 12\pi \sum_{i\in A} \sum_{j\in B} \sum_{k\in C} \left(P_{ij}P_{jk}P_{ki} - P_{ik}P_{kj}P_{ji} \right), \tag{S1}$$

where P is the projection operator onto the eigenmodes below the cut-off energy, A, B, C are three spatial regions shown in Fig. S3 and i, j, k are sites within corresponding regions.

The summation region is fixed to span half of the side length of the lattice, as shown by the square in Fig. S3, and it is divided into three regions A, B, and C represented by different colors. To study the phase transition under different hopping phases $e^{-i\phi}$, the cut-off frequency is swept, and the topological index is averaged over 20 random realizations of the amorphous graph. The phase diagram in Fig. 2 of the main text shows that a Kitaev sum of ± 1 can be reached by tuning the hopping phase. As an example, the standard deviation of the Kitaev sum with phase $\phi = \pi/2$ is shown in Fig. S3b, revealing that the Kitaev sum remains -1 within the topological bandgap with negligible variance across realizations of structural disorder. As a comparison, the Kitaev sum of a similar periodic system is show in Fig. S3c. The periodic phase diagram has sharper boundaries due to lack of the localized bulk modes near the band edges. The bandgap closes for phase values of $\phi = 0$, $1/3\pi$, $2/3\pi$ and π, in agreement with Fig. S1b.

S.4. ROBUSTNESS TO ON-SITE DISORDER

Despite the structural disorder, the amorphous system is robust to on-site (potential) disorder, up to a certain strength. In actual physical systems, such disorder may stem from fabrication imperfections that e.g. shift the natural frequency of the resonators employed as lattice sites. The on-site disorder is assumed here to follow a Gaussian distribution, characterized by the standard deviation σ which we sweep for the numerical simulations. The existence of a transport channel along the topological edge state is probed at the output site, with a single frequency excitation on the input port. The transport can be visualized by the intensity distribution at the steady state, shown in Fig. S4a-c. As the strength of on-site disorder increases, the edge state couples to localized modes near the boundary and the transport efficiency is reduced to half when the standard deviation of the on-site disorder is equal to the coupling strength between sites, κ. As shown in Fig. S4d, h, the transmission is further reduced to less than 5% as the disorder strength reaches 3κ.

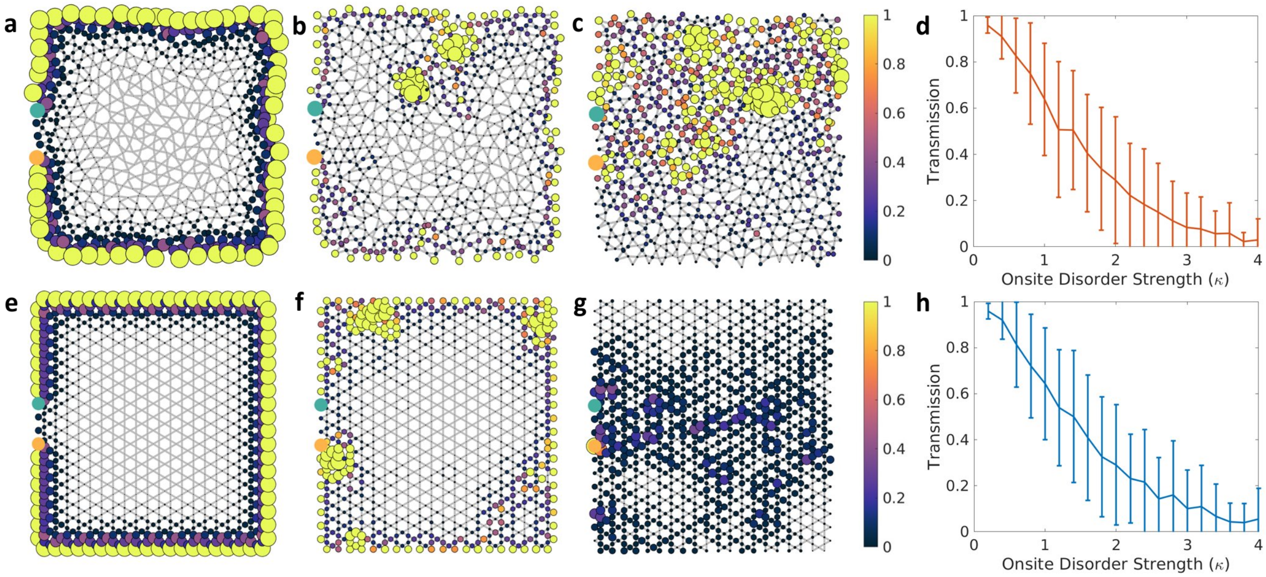

Fig. S4 | Robustness to on-site disorder. a–c, Topological transport on an amorphous graph with different on-site potential disorder. The disorder strength is characterized by the standard deviation of a Gaussian distribution with $\sigma = 0$ (**a**), 0.4 (**b**), 1.6 (**c**). The transmission with increasing on-site potential is summarized in **d** by averaging over 100 realizations of disorder. **e–h**, Topological transport on a periodic graph with potential disorder, similar to **a–c**.

S.5. PHOTONIC IMPLEMENTATION PARAMETERS

We envision a potential implementation of an amorphous topological graph on a silicon nitride photonic platform.[29] The photon pair generation efficiency and the corresponding timescale are then expressed in relation to the coupling strength between adjacent sites. In practice, we assume a coupling strength of $\kappa = 40\,\text{GHz}$, which translates to a timescale of $\kappa^{-1} = 0.025\,\text{ns}$. For the typical graph sizes used in our simulation, the time it takes for the light to

travel from the input to the output port is roughly $T = 1000\kappa^{-1} = 25\,\text{ns}$. For a typical single-mode silicon nitride waveguide, the effective area is of the order of $A = 0.5\,\mu\text{m} \cdot 0.2\,\mu\text{m} = 10^{-13}\,\text{m}^2$. For a pump power 1 mW, the intensity inside the waveguide is $I = 10^{10}\,\text{W m}^{-2}$. With a Kerr coefficient of silicon nitride around $n_2 = 10^{-18}\,\text{W}^{-1}\,\text{m}^2$, the nonlinear effect can be quantified by the relative change of refractive index as $\delta n/n = 10^{-5}$ within a ring resonator with quality factor of order 10^3. The corresponding frequency shift of the resonance of $n_2 I/\kappa = 0.1$ is used in the simulations with the normalized nonlinear coefficient $U/\kappa = 0.03$ and the coupling coefficient of the input coupler as $\kappa_{ex} = \kappa^2/2$.

S.6. TIME-DOMAIN FOUR-WAVE MIXING SIMULATION

In the spontaneous four-wave mixing process, signal and idler photon pairs are generated and transported along the chiral edge states. In practice, e.g. in a microrings platform, the generated photon pairs can be hosted at different resonance frequencies of the ring resonators.[29] In this case, the frequencies of the generated light differ from the pump frequency by a free spectral range, so that the pump light can be filtered out. The Hamiltonian of signal and idler frequencies in the linear regime is assumed to be the same as the pump, which have chiral transport channels along the edge, shown in Fig. 2 of the main text. The four-wave mixing Hamiltonian can be represented as[31]

$$H_{SI} = \begin{bmatrix} H_S & C \\ C^\dagger & H_I^\dagger \end{bmatrix}, \tag{S2}$$

where $H_S = H_I = H_0$ and $C_{ij} = \chi_i(t)\delta_{ij}$. Here, the four-photon interaction strength at site i, represented as $\chi_i(t)$, is time dependent, and proportional to the intensity of the pump at site i. The linear dynamics of signal and idler frequencies are governed by the diagonal part of the Hamiltonian, while the pump-induced photon pair generation is represented as the off-diagonal component. The four-wave mixing process is simulated in the time domain under the undepleted pump assumption, as the pump experiences self-modulation, but it is not affected by signal or idler photons due to their relatively low intensity. To quantify the photon pair generation efficiency, a small signal at idler frequency is injected into the system, and the signal at the output port is recorded and Fourier-transformed to obtain the spectral information. The evolution of the generated signal photon spectrum over time is calculated by a short-time Fourier transform of the output signal. A snapshot of the field distribution in the amorphous system is shown in Fig. S5, where the pump, idler and signal field profiles are plotted in panels a, b, c, respectively. In the photon pair generation process, the momentum and energy conservation has to be satisfied. In the amorphous system, the effective momenta of the excited edge modes can be estimated by calculating the phase differences between adjacent sites along the edge, which are visualized as zoomed-in views in Fig. S5d–f. The accumulated phase differences are plotted against the site index in Fig. S5g, from which we can extract the average phase shift per site as $\Delta\phi_p = -0.293$, $\Delta\phi_i = -0.583$, $\Delta\phi_i = -0.012$. The momentum conservation can then be verified in terms of the phase differences as $2\Delta\phi_p = \Delta\phi_s + \Delta\phi_i$, which is a natural consequence of the four-wave mixing simulation.

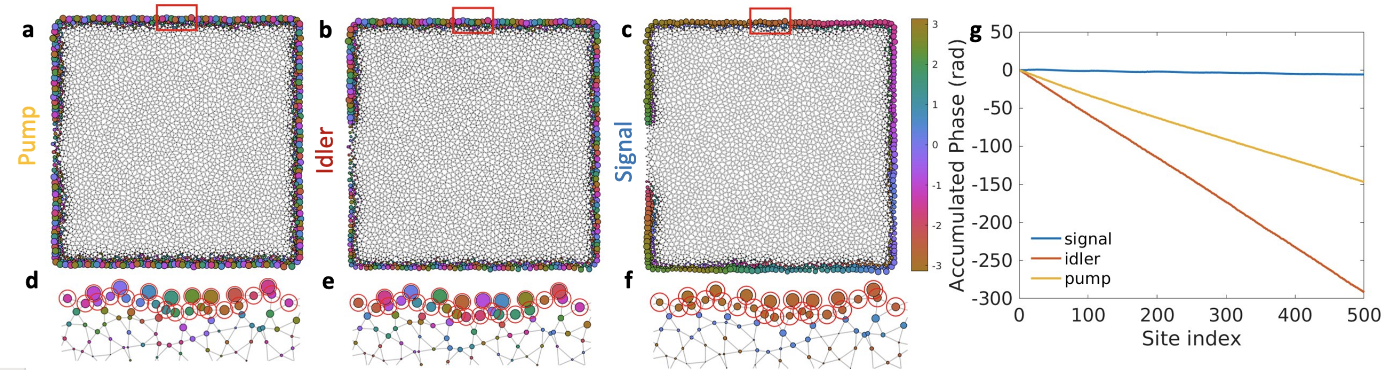

Fig. S5 | Four-wave mixing field profiles. The light is injected into the input port and coupled out at the output port, similar to Fig. 3 of the main text. **a**–**c**, Field profiles for pump (**a**), idler (**b**), and signal (**c**), where the color (size) represents the phase (amplituede) at each site. **d**–**f**, Zoomed-in view of the field distribution at sites along the edge. Red circles indicate the sites used for the phase calculation in **g**. **g**, Accumulated phases over 500 edge sites for pump, idler and signal fields.

S.7. DIFFERENT AMORPHOUS REALIZATIONS AND TRUNCATIONS

In the main text, we show the generated amorphous topological graph, following the generation procedure described in Sec. S.2 with a square boundary shape. Here, we show that the enhanced transport in amorphous system is independent of the specific realization of structural disorder, and is not limited to a square boundary truncation. As an example, we implement different realizations of the amorphous graphs, and compare them with the periodic systems truncated at different orientations. For each system, the same signal is injected into the structure and the short-time Fourier transform is computed, in order to capture the frequency information of the lattice intensity over time. For the amorphous graph, the short-time Fourier transform is similar to the square cut in Fig. 3 of the main text, as different truncations of the graphs have negligible effect on the amorphous system. The periodic system, however, shows different response under different truncation orientations. Nevertheless, the energy confined in the edge state of the amorphous system decays at a much slower rate compared to the periodic system, independently of the truncation choice.

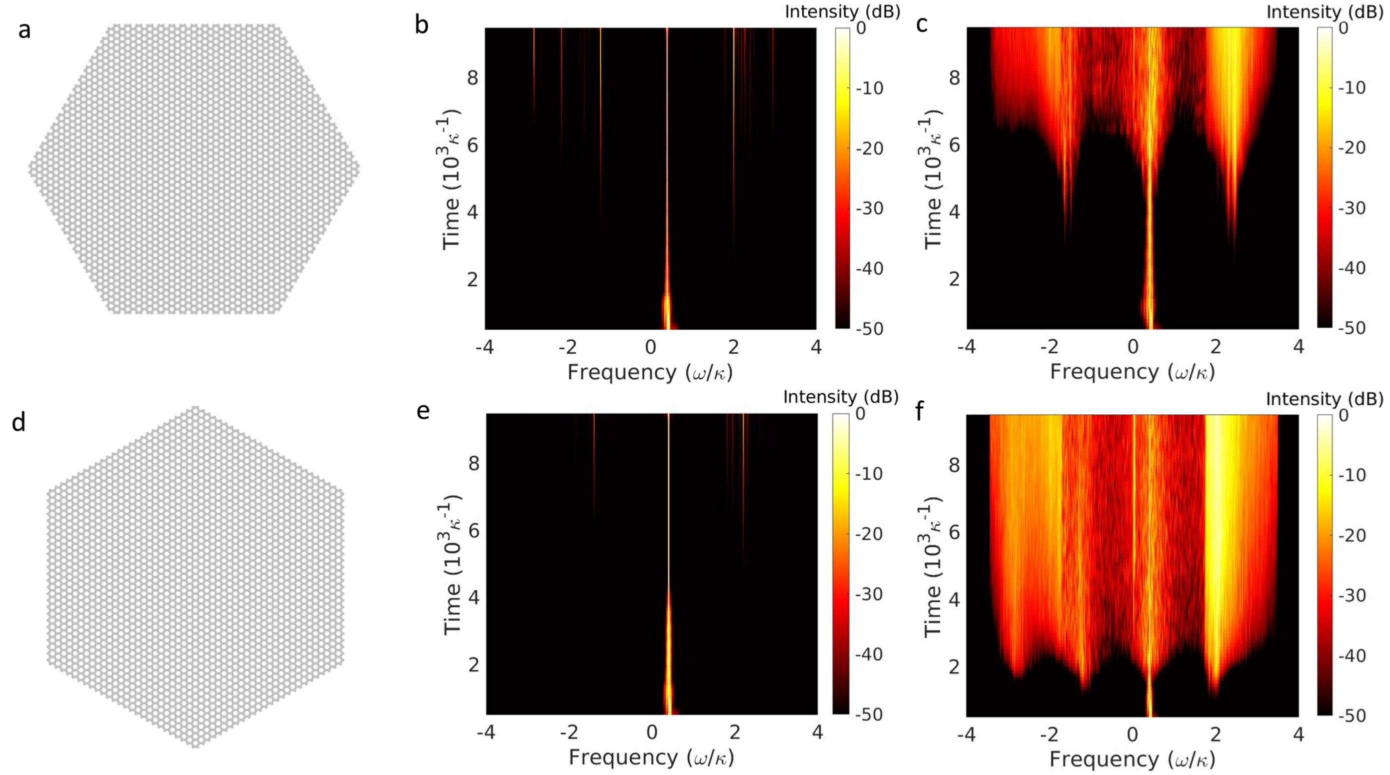

Fig. S6 | Different realizations and truncations. The periodic system is truncated along different lattice orientation directions, and the short-time Fourier transform of the signal is compared to the amorphous system. **a**, Armchair-like truncation of the periodic lattice. **b**, Short-time Fourier transform of energy transported in an amorphous system with nonlinearity. **c**, Short-time Fourier transform for transport in the periodic system. **d**–**f**, Same as **a**–**c**, but with a zig-zag-like truncation.

REFERENCES AND NOTES

1. D. J. Thouless, M. Kohmoto, M. P. Nightingale, M. den Nijs, Quantized Hall conductance in a two-dimensional periodic potential. *Phys. Rev. Lett.* **49**, 405–408 (1982).

2. L. B. Ioffe, M. V. Feigel'man, A. Ioselevich, D. Ivanov, M. Troyer, G. Blatter, Topologically protected quantum bits using Josephson junction arrays. *Nature* **415**, 503–506 (2002).

3. K. V. Klitzing, G. Dorda, M. Pepper, New method for high-accuracy determination of the fine-structure constant based on quantized hall resistance. *Phys. Rev. Lett.* **45**, 494–497 (1980).

4. D. C. Tsui, H. L. Stormer, A. C. Gossard, Two-dimensional magnetotransport in the extreme quantum limit. *Phys. Rev. Lett.* **48**, 1559–1562 (1982).

5. B. Huckestein, Scaling theory of the integer quantum Hall effect. *Rev. Mod. Phys.* **67**, 357–396 (1995).

6. A. C. M. de Oca, D. Martinez-Pedrera, Role of impurities in stabilizing quantum Hall effect plateaus. *Phys. Rev. B* **67**, 245310 (2003).

7. J. Moore, The birth of topological insulators. *Nature* **464**, 194–198 (2010).

8. Q. L. He, T. L. Hughes, N. P. Armitage, Y. Tokura, K. L. Wang, Topological spintronics and magnetoelectronics. *Nat. Mater.* **21**, 15–23 (2022).

9. Z. Wang, Y. D. Chong, J. D. Joannopoulos, M. Soljačić, Reflection-free one-way edge modes in a gyromagnetic photonic crystal. *Phys. Rev. Lett.* **100**, 013905 (2008).

10. M. Hafezi, E. A. Demler, M. D. Lukin, J. M. Taylor, Robust optical delay lines with topological protection. *Nat. Phys.* **7**, 907–912 (2011).

11. S. Mittal, J. Fan, S. Faez, A. Migdall, J. M. Taylor, M. Hafezi, Topologically robust transport of photons in a synthetic gauge field. *Phys. Rev. Lett.* **113**, 087403 (2014).

12. P. St-Jean, V. Goblot, E. Galopin, A. Lemaître, T. Ozawa, L. le Gratiet, I. Sagnes, J. Bloch, A. Amo, Lasing in topological edge states of a one-dimensional lattice. *Nat. Photonics* **11**, 651–656 (2017).

13. B. Bahari, A. Ndao, F. Vallini, A. el Amili, Y. Fainman, B. Kanté, Nonreciprocal lasing in topological cavities of arbitrary geometries. *Science* **358**, 636–640 (2017).

14. B. Bahari, L. Hsu, S. H. Pan, D. Preece, A. Ndao, A. el Amili, Y. Fainman, B. Kanté, Photonic quantum Hall effect and multiplexed light sources of large orbital angular momenta. *Nat. Phys.* **17**, 700–703 (2021).

15. H. Zhao, X. Qiao, T. Wu, B. Midya, S. Longhi, L. Feng, Non-Hermitian topological light steering. *Science* **365**, 1163–1166 (2019).

16. D. Smirnova, D. Leykam, Y. Chong, Y. Kivshar, Nonlinear topological photonics. *Appl. Phys. Rev.* **7**, 021306 (2020).

17. D. Weaire, Existence of a gap in the electronic density of states of a tetrahedrally bonded solid of arbitrary structure. *Phys. Rev. Lett.* **26**, 1541–1543 (1971).

18. D. Weaire, M. F. Thorpe, Electronic properties of an amorphous solid. I. A simple tight-binding theory. *Phys. Rev. B* **4**, 2508–2520 (1971).

19. A. Agarwala, V. B. Shenoy, Topological insulators in random lattices. *Phys. Rev. Lett.* **118**, 236402 (2017).

20. H. Huang, F. Liu, Quantum spin Hall effect and spin Bott index in a quasicrystal lattice. *Phys. Rev. Lett.* **121**, 126401 (2018).

21. M. A. Bandres, M. C. Rechtsman, M. Segev, Topological photonic quasicrystals: Fractal topological spectrum and protected transport. *Phys. Rev. X* **6**, 011016 (2016).

22. P. Zhou, G.G. Liu, X. Ren, Y. Yang, H. Xue, L. Bi, L. Deng, Y. Chong, B. Zhang, Photonic amorphous topological insulator. *Light Sci. Appl.* **9**, 133 (2020).

23. N. P. Mitchell, L. M. Nash, D. Hexner, A. M. Turner, W. T. M. Irvine, Amorphous topological insulators constructed from random point sets. *Nat. Phys.* **14**, 380–385 (2018).

24. Q. Marsal, D. Varjas, A. G. Grushin, Topological Weaire-Thorpe models of amorphous matter. *Proc. Natl. Acad. Sci. U.S.A.* **117**, 30260–30265 (2020).

25. D. R. Hofstadter, Energy levels and wave functions of Bloch electrons in rational and irrational magnetic fields. *Phys. Rev. B* **14**, 2239–2249 (1976).

26. F. D. M. Haldane, Model for a quantum Hall effect without Landau levels: Condensed-matter realization of the “parity anomaly”. *Phys. Rev. Lett.* **61**, 2015–2018 (1988).

27. A. Kitaev, Anyons in an exactly solved model and beyond. *Ann. Phys. Rehabil. Med.* **321**, 2–111 (2006).

28. N. F. Mott, R. S. Allgaier, Localized states in disordered lattices. *Phys. Status Solidi B* **21**, 343–356 (1967).

29. S. Mittal, E. A. Goldschmidt, M. Hafezi, A topological source of quantum light. *Nature* **561**, 502–506 (2018).

30. S. Mittal, V. V. Orre, E. A. Goldschmidt, M. Hafezi, Tunable quantum interference using a topological source of indistinguishable photon pairs. *Nat. Photonics* **15**, 542–548 (2021).

31. J. R. Ong, S. Mookherjea, Quantum light generation on a silicon chip using waveguides and resonators. *Opt. Express* **21**, 5171–5181 (2013).

32. R. Kumar, J. R. Ong, M. Savanier, S. Mookherjea, Controlling the spectrum of photons generated on a silicon nanophotonic chip. *Nat. Commun.* **5**, 5489 (2014).

33. K. Ohgushi, S. Murakami, N. Nagaosa, Spin anisotropy and quantum Hall effect in the kagomé lattice: Chiral spin state based on a ferromagnet. *Phys. Rev. B* **62**, R6065–R6068 (2000).
